# Urinary proteome analysis of acute kidney injury in post-cardiac surgery patients using enrichment materials with high-resolution mass spectrometry

**DOI:** 10.3389/fbioe.2022.1002853

**Published:** 2022-09-13

**Authors:** Yunpeng Bai, Ying Li, Zhizhong Tang, Linhui Hu, Xinyi Jiang, Jingchun Chen, Sumei Huang, Kunyong Wu, Wang Xu, Chunbo Chen

**Affiliations:** ^1^ Center of Scientific Research, Maoming People’s Hospital, Maoming, China; ^2^ Department of Critical Care Medicine, Maoming People’s Hospital, Maoming, China; ^3^ Department of Intensive Care Unit of Cardiovascular Surgery, Guangdong Cardiovascular Institute, Guangdong Provincial People’s Hospital, Guangdong Academy of Medical Sciences, Guangzhou, China; ^4^ Department of Urology, Maoming People’s Hospital, Maoming, China; ^5^ School of Medicine, South China University of Technology, Guangzhou, China; ^6^ Department of Emergency, Maoming People’s Hospital, Maoming, China; ^7^ Biological Resource Center of Maoming People’s Hospital, Maoming, China; ^8^ Department of Critical Care Medicine, Guangdong Provincial People’s Hospital, Guangdong Academy of Medical Sciences, Guangzhou, China; ^9^ The Second School of Clinical Medicine, Southern Medical University, Guangzhou, China; ^10^ Guangdong Provincial Key Laboratory of Renal Failure Research, Southern Medical University, Guangzhou, China

**Keywords:** urinary proteome, acute kidney injury, high-resolution mass spectrometry, differential analysis, gemini C18

## Abstract

**Background:** Cardiac surgery-associated acute kidney injury (CSA-AKI) may increase the mortality and incidence rates of chronic kidney disease in critically ill patients. This study aimed to investigate the underlying correlations between urinary proteomic changes and CSA-AKI.

**Methods:** Nontargeted proteomics was performed using nano liquid chromatography coupled with Orbitrap Exploris mass spectrometry (MS) on urinary samples preoperatively and postoperatively collected from patients with CSA-AKI. Gemini C18 silica microspheres were used to separate and enrich trypsin-hydrolysed peptides under basic mobile phase conditions. Differential analysis was conducted to screen out urinary differential expressed proteins (DEPs) among patients with CSA-AKI for bioinformatics. Kyoto Encyclopedia of Genes and Genomes (KEGG) database analysis was adopted to identify the altered signal pathways associated with CSA-AKI.

**Results:** Approximately 2000 urinary proteins were identified and quantified through data-independent acquisition MS, and 324 DEPs associated with AKI were screened by univariate statistics. According to KEGG enrichment analysis, the signal pathway of protein processing in the endoplasmic reticulum was enriched as the most up-regulated DEPs, and cell adhesion molecules were enriched as the most down-regulated DEPs. In protein–protein interaction analysis, the three hub targets in the up-regulated DEPs were α-1-antitrypsin, β-2-microglobulin and angiotensinogen, and the three key down-regulated DEPs were growth arrest-specific protein 6, matrix metalloproteinase-9 and urokinase-type plasminogen activator.

**Conclusion:** Urinary protein disorder was observed in CSA-AKI due to ischaemia and reperfusion. The application of Gemini C18 silica microspheres can improve the protein identification rate to obtain highly valuable resources for the urinary DEPs of AKI. This work provides valuable knowledge about urinary proteome biomarkers and essential resources for further research on AKI.

## Introduction

As a serious life-threatening syndrome, acute kidney injury (AKI) can lead to the imbalance of renal excretory metabolites and the impaired regulation of electrolytes and acid–base balance (Lameire et al., 2013). Given that AKI may be caused by many reasons (such as major surgery, ischaemia, sepsis and drugs), it has a relatively high incidence among patients in intensive care units and has become a common complication in critically ill patients (Santos et al., 2019). Although great progress has been achieved in the supportive therapy of AKI, this condition is still diagnosed following the previous standards: an increase in serum creatinine (sCr) and a decrease in urine volume (Kellum and Lameire., 2013). However, both are not the best early diagnostic indicators of AKI because they may frequently change after renal dysfunction (Bennett and Devarajan., 2011). Therefore, early AKI diagnosis has become one of the research hotspots in recent years.

As a sensitive indicator of renal glomerular and tubular injury, the quantitative proteinuria spectrum has always been important in predicting kidney injury. Some AKI protein biomarkers with potential clinical value have been recently investigated, including the serum/urine levels of cystatin C to reflect the glomerular filtration rate (Liang et al., 2020; Deng et al., 2019; Hou et al., 2021; Deng et al., 2020; Zhang et al., 2019) and the relationship between renal tubular epithelial cell damage and inflammatory factors, such as kidney injury molecule-1, liver type fatty acid binding protein and neutrophil gelatinase-associated lipocalin (Hu et al., 2022; Liang et al., 2022; Zdziechowska et al., 2020). Previous studies on proteomics and early pathobiological events demonstrated that urinary angiotensinogen (AGT) and urinary matrix metalloproteinase-7 (MMP-7) could be used as AKI biomarkers for clinical applications (Chen C et al., 2016; Yang et al., 2017).

Urine contains the whole excreted products from the kidneys, so the proteomic changes in renal disease can be reflected by the urinary proteomic profiles of patients (Kim et al., 2011). As a non-invasive source, urine samples can be readily obtained in large quantities with expected participant compliance and a low risk of infection to researchers (Bai et al., 2022). Thus, urinary profile analysis is an attractive option for the discovery of proteomic biomarkers, especially for the diagnosis of renal diseases and the molecular mechanisms of renal pathology (Nkuipou-Kenfack et al., 2020). For example, steroid-resistant and steroid-sensitive nephrotic syndromes can be distinguished by urinary proteome for paediatric patients (Khurana et al., 2006). Additionally, the aetiology and pathogenesis of AKI are extremely complex due to the highly heterogeneous clinical syndrome for severe patients, so a single biomarker cannot comprehensively and accurately reflect the overall pathophysiological changes of renal injury (Liang et al., 2012). Therefore, highly sensitive and specific urinary proteomic spectra can help improve the diagnosis, classification and treatment of AKI.

Given that AKI may be involved in many different types of molecules with a large dynamic concentration range, the pre-treatment of urine samples is an important step in the study of low-abundance protein biomarkers, especially when using functional materials with separation and enrichment functions. Porous polymer materials can separate target molecules from complex matrices and can be applied in various fields, from liquid purification to biomolecular fractionation (Liu et al., 1999). Heterogenous pore polymer particles, synthesised by double emulsion interfacial polymerisation, can effectively enrich the low-abundance glycopeptides (Song et al., 2018) and rapidly separate the proteins with similar sizes (Song et al., 2019). In this study, we performed the comparative proteomic analysis of urine samples from six patients with cardiac surgery-associated (CSA)-AKI using Gemini C18 column and high-resolution mass spectrometry (HRMS). The results of this urinary proteome study may help clarify the mechanisms of CSA-AKI and the application of enrichment materials in bioseparation. This work also provides valuable knowledge about urinary proteome biomarkers.

## Materials and methods

### Chemicals and reagents

MS-grade acetonitrile (ACN) was purchased from Thermo Fisher Scientific, United States, and ultrapure water was filtered through the Milli-Q system (Millipore, Billerica, MA). Formic acid (FA) was obtained from DIMKA, and ammonium bicarbonate was purchased from Fluka (Honeywell Fluka, United States). Sodium dodecyl sulfate (SDS), SDS-free protein lysate, dithiothreitol (DTT), iodoacetamide (IAM), Coomassie brilliant blue G-250, trypsin and acetone were obtained from Aladdin, China.

### Patients and urinary proteins extraction

This investigation was carried out according to the World Medical Association Declaration of Helsinki and approved by the ethics committee of Maoming People’s Hospital. All patients had completed written informed consents before cardiac surgery, and six patients were enrolled in the urinary proteomics study after the diagnosis of AKI which was defined in accordance with the KDIGO Clinical Practice Guidelines based on sCr criteria: the increase in sCr ≥ 50% within 7 days, or the increase in sCr ≥ 0.3 mg/dl (≥ 26.5 μmol/L) within 48 h, or oliguria (Khwaja, 2012). Patients were excluded if they were age under 18 years, had preexisting chronic renal disease or undergoing renal replacement therapy, a history of renal transplantation or nephrectomy. In order to explore the effect of CSA-AKI on kidney, spot urine samples were prospectively collected at two time points, preoperative (Before_AKI) and postoperative-the first day of diagnosed CSA-AKI (AKI-Day1), which was used to compare the changes in urinary proteomic profiles. Serum creatinine was measured before cardiac surgery, at least twice a day for the first 3 days after cardiac surgery, and then daily thereafter.

Approximate 5 ml of urine sample from Biological Resource Center of Maoming People’s Hospital, was transferred into a 50 ml centrifuge tube, and 25 ml (5 times the volume of urine sample) of precooled acetone was added into the tube. After placing at -20°C overnight, the urine samples were centrifuged at 25,000 *g* and 4°C for 15 min, and the supernatant of each sample were discarded. Then, an appropriate amount of SDS-free protein lysate was added into the drying urine sample, and an automatic grinder was used to promote protein dissolution. The supernatants were collected after centrifuging at 25,000 *g* at 4°C for another 15 min. Final concentration (10 mM) of DTT was added into the supernatant and bathed in water at 37°C. After 30 min water bath, final concentration (55 mM) of IAM was added into the reduced samples and kept for another 45 min in the dark at room temperature. Protein solution could be obtained from the supernatant of alkylated samples by centrifuging at 25,000 *g* at 4°C for 15 min. At last, the Bradford assay kit (Bio-Rad, Hercules, CA, United States) was used to determine the concentration of the protein solution.

### Protein enzymatic digestion and high pH column separation

As for protein enzymatic digestion, 100 μg of protein solution per sample was transferred and diluted with 4 times volumes of 50 mM NH_4_HCO_3_ solution. According to the ratio of 40: 1 (protein to enzyme), 2.5 μg of trypsin was added into the diluted protein solution for enzymatic digestion at 37°C. After 4 h, enzymatic peptides were desalted using a Strata X column and vacuumed to dryness.

In Shimadzu LC-20AB high performance liquid chromatography (HPLC) system, the Gemini C18 00G-4435-E0 column (5 μm, 4.6 mm internal diameter×250 mm length) was used for peptide separation with mobile phase A (5% ACN, pH 9.8) and mobile phase B (95% ACN). Equal amount of peptides were mixed from all urinary proteome samples, which was further diluted with mobile phase A and eluted at a flow rate of 1 ml/min by the following gradient: 0–10 min 95% A, 5% B; 10–50 min, 95%–65% A, 5%–35% B; 50–51 min 65%–5% A, 35%–95% B; 51–54 min, 5%–0% A, 95%–100% B; finally 95% A and 5% B eluting 10 min to re-equilibrate the column. The elution peaks were monitored at 214 nm and the fractions were collected every minute. At last, the peptide components were combined into a total of 10 fractions for lyophilization and Data Dependent Acquisition (DDA) analysis.

### Characterization of gemini C18 silica microspheres

The morphology of Gemini C18 silica microspheres was characterized by a JSM-6700F scanning electron microscope (SEM, JEOL, Japan) at 3.0 kV, and element analysis was performed on an energy dispersive spectrometer (EDS) of the SEM. The particle size distribution was measured by ImageJ software through SEM image, and the zeta potential of the particles (dispersed in ethanol) was measured by a nanosize and zeta potential analyzer (Malvern, United Kingdom). The water contact angles (CAs) on the coating composed of the silica microspheres were measured on a CA system (OCA20, Dataphysics, Germany) at ambient temperature, and the average CA value was obtained by measuring three different positions of the particles.

### Urinary proteome analysis by nano-LC-MS/MS

An Orbitrap Exploris 480 (Thermo Fisher Scientific, San Jose, CA) system equipped was used to analyze urinary proteome for peptide sequencing and protein quantification. A Thermo Ultimate 3000 UHPLC liquid chromatography with the trap column and a tandem self-packed C18 column (150 μm internal diameter, 1.8 μm of silica microspheres diameter, 35 cm column length), were coupled online to the mass spectrometer through a nanoESI ion source.

DDA Library Construction: The segmented dried peptide samples were reconstituted with mobile phase A (2% ACN, 0.1% FA aqueous solution), and the supernatants were collected for injection after centrifuging at 20,000 *g* for 10 min. Enriched peptides were separated at a flow rate of 500 nL/min by the following effective gradient for DDA mode detection: 0–5 min, 5% mobile phase B (98% ACN, 0.1% FA); 5–120 min, 5%–25% B; 120–160 min, 25%–35% B; 160–170 min, 35%–80% B; 170–175 min, 80% B; 175–175.5 min, 80%–5% B; 175.5–180 min, 5% B. The main parameters of Orbitrap MS were set as follows: ion source voltage 1.9kV; MS1 scanning range *m/z* 350∼1,650; MS1 resolution 120,000; maximal injection time (MIT) 90 ms; MS/MS collision type HCD; collision energy NCE 30; MS/MS resolution 30,000; auto mode for MIT; dynamic exclusion duration 120 s. The start *m/z* for MS/MS was fixed to auto mode, and AGC was set as MS 300% and MS/MS 100%. Precursor peptide ions for MS/MS scan were satisfied: charge range from 2 + to 6+, top 30 precursors with intensity over 2E4.

Data Independent Acquisition (DIA) Protein Quantification: The dried enzymatic urinary proteome samples were reconstituted with mobile phase A (2% ACN, 0.1% FA aqueous solution) and centrifuged at 20,000 *g* for 10 min. The supernatants were injected into the nanoLC-MS system and separated at a flow rate of 500 nL/min by the following gradient program: 0–5 min, 5% mobile phase B (98% ACN, 0.1% FA); 5–90 min, 5%–25% B; 90–100 min, 25%–35% B; 100–108 min, 35%–80% B; 108–113 min, 80% B; 113–113.5 min, 80%–5% B; 113.5–120 min, 5% B. The main parameters of MS in DIA mode were set as follows: ion source voltage 1.9 kV, MS1 scanning range *m/z* 400∼1,250; MS1 resolution 120,000; MIT 90 ms. For MS/MS scan, the scanning range *m/z* 400∼1,250 was equally divided to 50 continuous windows, and fragment ions were scanned in Orbitrap with resolution of 30,000. MS/MS collision type was also selected as HCD, collision energy NCE was set to 30, MIT was auto mode, and AGCs were separately set as MS 300% and MS/MS 1000%.

### Protein identification and quantitative analysis

In the DDA library construction, the software MaxQuant (version 1.5.3.30) was used for peptide identification, and the retrieved database was uniprot_homosapiens_irt.fasta (20,303 sequences). Trypsin was used for proteins cutting and up to two missed cuts were allowed. Methyl carbamate was used for the fixed modification at cysteine (C) site, while oxidized of methionine (M), acetyl groups (N-terminal of protein), glutamine (Q) to pyro-glutamate (N-terminal of Q) and deamidated (NQ) were used for variable modification. The error rate for peptide mapping matching with protein was set as 1%, and the shortest peptide length was seven amino acids. The DIA MS data were analyzed by the software Spectronaut, and the retention time (RT) was corrected by iRT peptides. Based on the target-decoy model suitable for sequential window acquisition of theoretical fragment ion mass spectrometry (SWATH-MS), the false positive control was set as 1%. Finally, the significant differences of urinary proteome were statistically evaluated by the MSstats software package. After error correction and normalization were performed on each sample, the differential expressed proteins (DEPs) were screened out according to the absolute value of fold change (|FC|) ≥ 2 and *p* value < 0.05 as the judgment criteria for significant differences.

### Bioinformatic and statistical analysis

Gene ontology (GO) annotation of urinary proteome was analyzed using the online Database for Annotation, Visualization and Integrated Discovery (DAVID) software. The software Cluster 3.0 was used for hierarchical clustering and visualization on the urinary DEPs. Kyoto Encyclopedia of Genes and Genomes (KEGG) is a database collection referring to genomes, diseases, biological pathways, drugs and chemical materials, which was used for pathway analysis of the identified urinary proteins using the online software. The possible functions of urinary proteins were predicted by Eukaryoyic Orthologous Groups (KOG) software, and the online WoLF PSORT software was used to predict the possible subcellular locations of proteins. Finally, Search Tool for the Retrieval of Interacting Genes (STRING, 11.0) software was used to analyze the protein-protein interaction (PPI) network, which is important to understand cell physiology under normal and disease conditions.

## Results

### Characterisation of patients and silica particles

The basic information of six patients with CSA-AKI, such as age, body mass index (BMI), sCr levels and urinary protein amounts, is displayed in Supplementary Table S1. The urine samples were collected from patients with CSA-AKI and divided into two groups: Before_AKI (uninjured kidney) and AKI_Day1 (injured kidney). The sCr levels and urinary protein amounts of the AKI_Day1 group were significantly increased compared with those of the Before_AKI group. According to the 2012 KDIGO clinical practice guideline, AKI can be divided into three stages based on sCr elevation: stage 1A as an absolute increase in sCr of 0.3 mg/dl (or 26.5 μmol/L) within 48 h, stage 1B as a 50% relative increase in sCr within 7 days, stage 2 as a 100% relative increase in sCr over a 7-days window of observation, and stage 3 as a 200% relative sCr increase within 7 days. In total, the severity of six AKI patients separately belonged to three patients in stage 1A, two patients in stage 1B, and one patient in stage 2.

**FIGURE 1:**
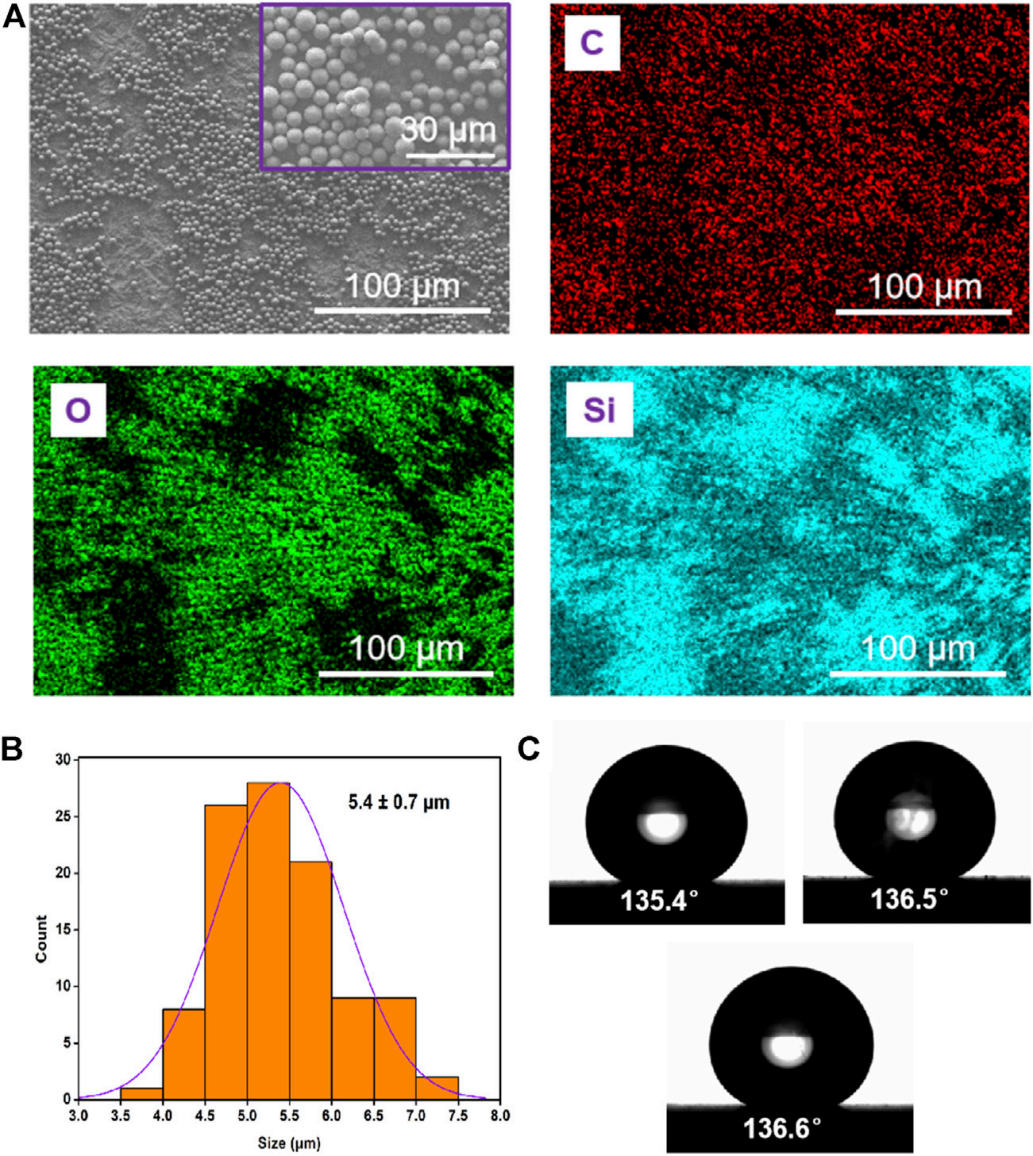
Characterisation of Gemini C18 silica microspheres. **(A)** SEM image and elemental analysis (C, O, and Si) of the particles. **(B)** Size distribution of the particles. **(C)** Photograph of a water droplet on the coating of silica microspheres surface; the water CA is about 136.1° ± 0.6°.

The high-magnification SEM image in Figure 1A showed the uniform particle size distribution of the silica microspheres. EDS mapping of the particles showed that carbon (C), oxygen (O) and silicon (Si) were only detected in the exterior region, suggesting the hydrophobicity of the surface of silica microspheres due to the grafted layer of alkane chains. The silica particles exhibited uniform size distribution with an average diameter of 5.4 ± 0.70 µm (mean ± SD, Figure 1B). The average zeta potential of these silica particles in ethanol was 13.70 ± 0.97 mV (three test values: 13.83, 12.73 and 14.53 mV), indicating that these particles could easily aggregate. Furthermore, the contact angle (CA, θ) of the three phases of the silica microspheres was measured to study their surface wettability (Figure 1C). The value of 136.1° ± 0.6° (mean ± SD, n = 3) was obtained, further proving the hydrophobic properties of the silica microsphere coating.

### Identification and quantification of urinary proteomes

DDA MS was used to identify and construct the peptide library of urinary proteomes from the Gemini column enriched samples (Supplementary Table S2). A total of 10,869 peptides and 1,942 proteins were identified from the preoperative and postoperative urine samples using DIA MS as listed in Supplementary Table S3 and Supplementary Table S4, respectively. The cysteine sites in the samples can be reduced and alkylated through the classical carbamidomethylated reaction with DTT and IAM prior to tandem MS analysis. A total of 1,781 peptide segments contained cysteines with carbamidomethylation. With the development of tandem MS, the number of posttranslational modifications (PTMs) identified on proteins has increased rapidly, providing valuable information on the signal pathways and cellular processes regulated by PTMs. In the study, the PTMs on the peptides included 961 oxidation of M, 105 acetylation of N-terminal, 218 of pyroglutamalytion on Q and 856 deamidation. The annotated data of the urinary proteomes of patients with AKI are listed in Supplementary Table S5.

The basic statistical results of the urinary proteins are shown in Supplementary Figure S1 and comprise unique peptide distribution, protein mass distribution and protein coverage distribution. According to the unique peptide distribution in Supplementary Figure S1A, 1,004 proteins had the number of unique peptides ≥ 3, accounting for 51.70% of all identified urinary proteins. Bar charts in Supplementary Figure S1B showed that the molecular masses of urinary proteins were mostly distributed in the range of 10–60 KDa. Therefore, most urinary proteins were medium proteins. For protein coverage distribution, the length of the identified sequence was divided by the total length of the protein sequence. Supplementary Figure S1C shows that the identification rate was mainly concentrated within the range of 40%.

**FIGURE 2:**
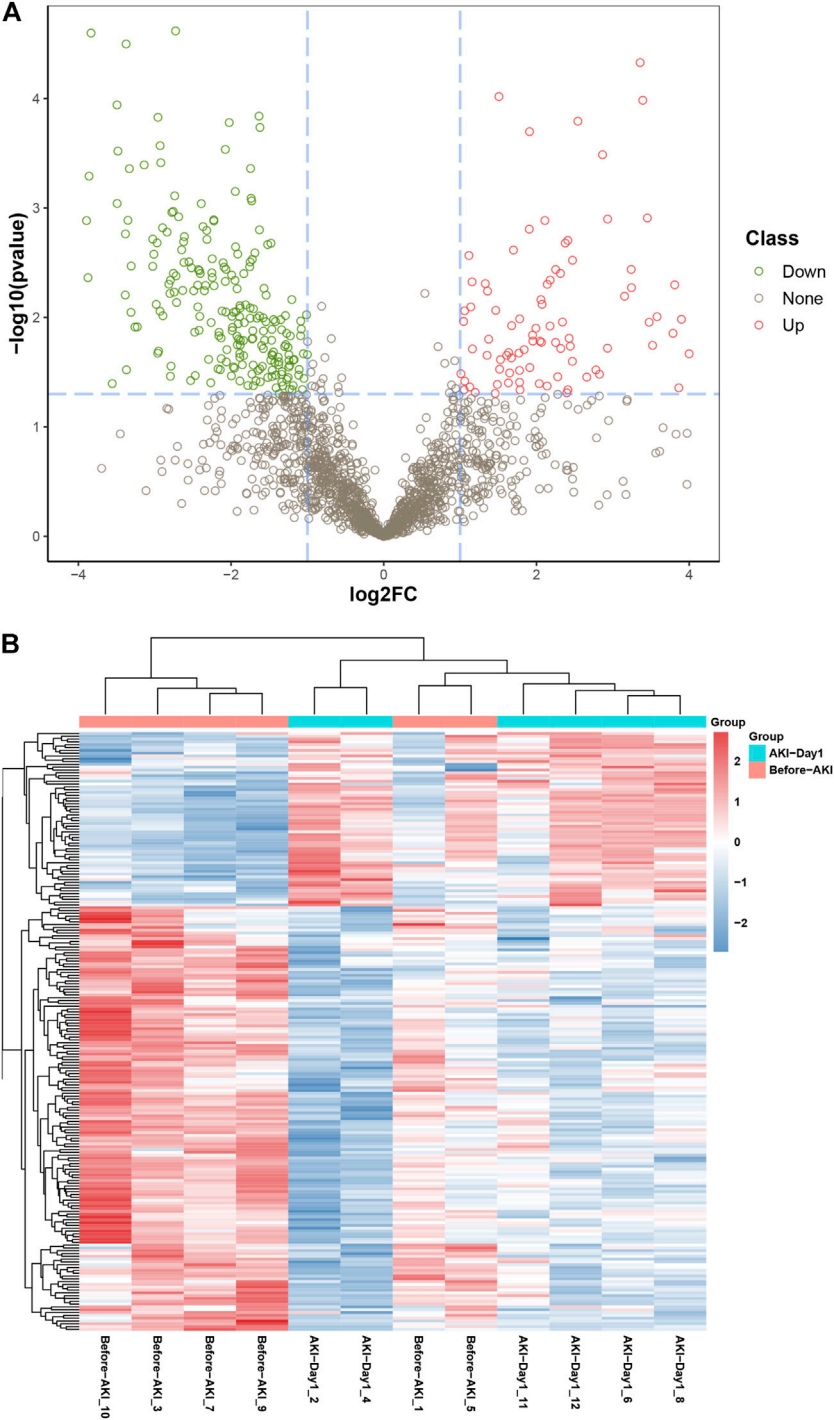
Differential expression of urinary proteins between groups. **(A)** Volcano plot for visually displaying the differential expressed proteins (DEPs): the red circles are the up-regulated DEPs, the green ones are the down-regulated DEPs, and the grey ones are the non-significant proteins. **(B)** Cluster analysis of DEPs from patients with AKI. The red part represents the proteins with high expression, and the blue part indicates the proteins with low expression.

The urinary proteome differences between groups were compared using univariate analysis to investigate the significant DEPs before and after AKI. The results were visualised through the volcano plot in Figure 2A. Changes in urinary proteins were determined by the combination of FC and *p* value; FC ≥ 2 and *p* value < 0.05 were expressed as up-regulation, and FC ≤ −2 and *p* value < 0.05 were expressed as down-regulation. Compared with the Before-AKI group, 324 proteins were differentially expressed in the AKI-Day1 group, with 96 up-regulated DEPs and 228 down-regulated DEPs. Furthermore, Figure 2B shows the cluster analysis chart of DEPs to visually reflect the expressed differences between the two groups.

### Gene ontology annotation and enrichment analysis

GO is an international standard gene function classification system with three categories: biological process (BP), cellular component (CC) and molecular function (MF). It can provide a timely updated standard vocabulary to comprehensively describe the characteristics of genes and gene products in organisms. Blast2GO software was used for GO annotation analysis to evaluate the functional significance of all identified proteins. As shown in Supplementary Figure S2A, the most enriched BP (out of 29 GO terms) were “cellular process”, “biological regulation”, “regulation of biological process”, “metabolic process” and “response to stimulus”. Meanwhile, the most enriched CC (out of 19 GO terms) were “cell”, “cell part”, “organelle”, “extracellular region” and “extracellular region part”. The most enriched MF (out of 13 GO items) were “binding”, “catalytic activity”, “molecular function regulator”, “molecular transducer activity” and “signal transducer activity”.

**FIGURE 3:**
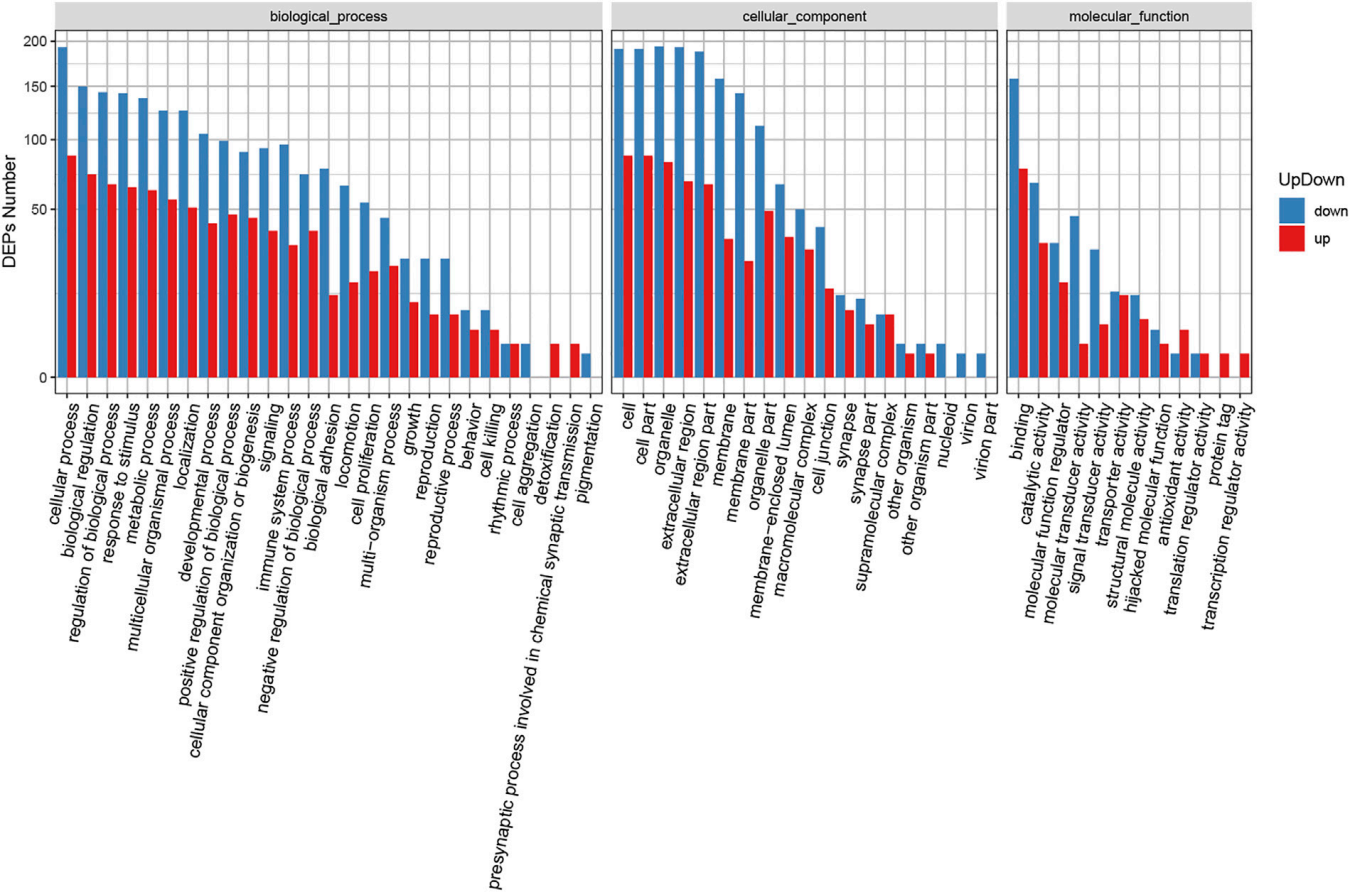
GO classification of up-regulated and down-regulated DEPs.

GO enrichment analysis was also carried out for the identified DEPs (Supplementary Table S6). Supplementary Figure S2B shows the GO functional classification maps of all DEPs, and Figure 3 displays the GO classification of up-regulated and down-regulated differential proteins. The up-regulated and down-regulated DEPs can interfere with common structural or functional processes. However, unique enrichments were detected for some GO terms, including the up-regulated proteins (e.g., detoxification, presynaptic processes involved in chemical synaptic transmission, protein tag and transcription regulator activity) and down-regulated proteins (e.g., cell aggregation, pigmentation, nucleoid, virion and virion part).

### Kyoto encyclopedia of genes and genomes enrichment analysis of differential expressed proteins

**FIGURE 4:**
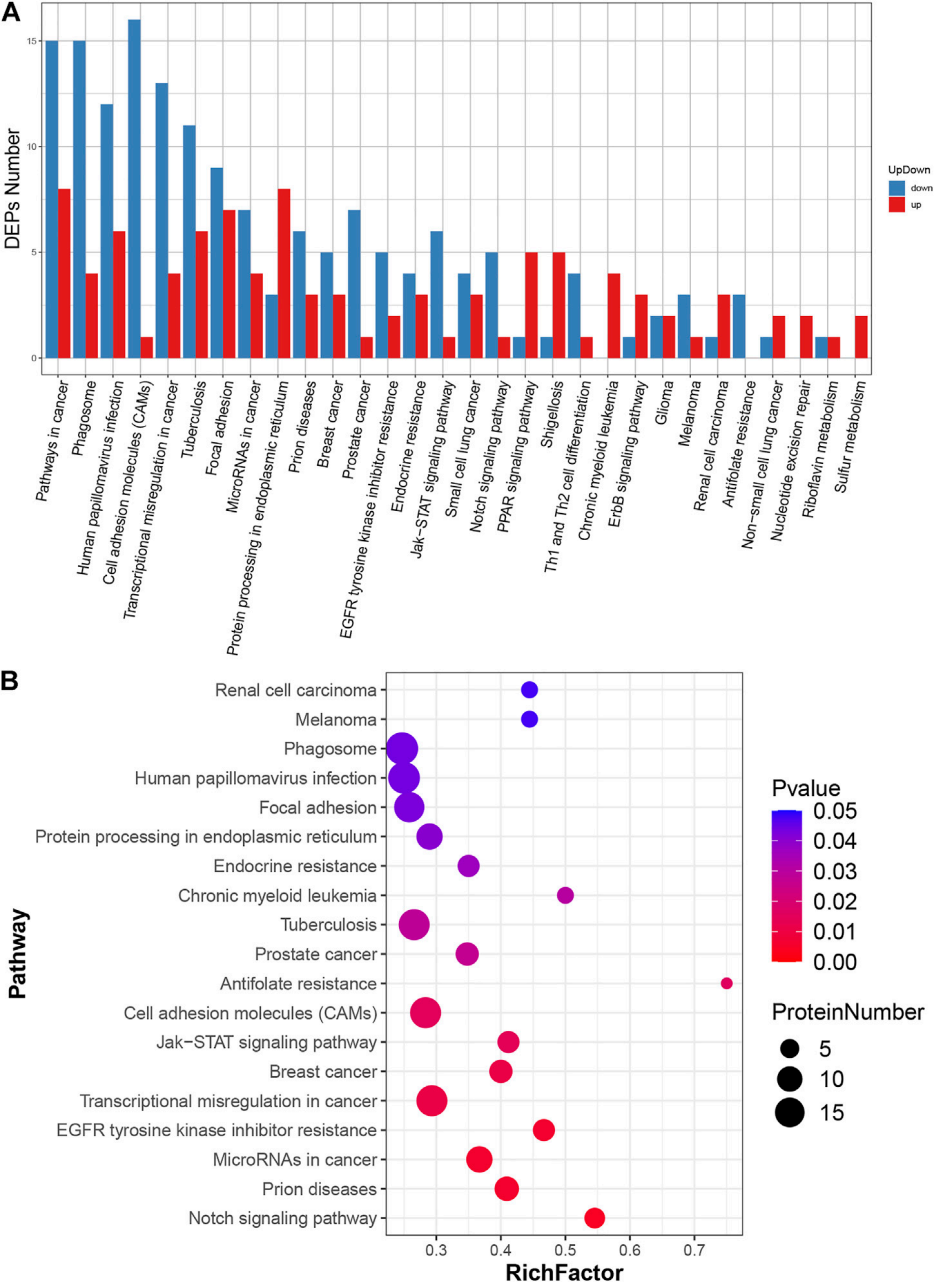
KEGG pathway analysis of DEPs. **(A)** Classification of DEPs. The x-axis represents pathway annotation entries, and the y-axis represents the number of DEPs enriched in each pathway term. **(B)** Significant enrichment pathways of DEPs. The size of the dot represents the number of DEPs annotated to the pathways, and the colour of the dot represents the *p*-value.

The biological functions of the identified DEPs were further characterised *via* KEGG enrichment analysis (Supplementary Table S7). The results showed that the up-regulated proteins were annotated as 29 major pathways and the most enriched pathways were “protein processing in endoplasmic reticulum” and “pathway in cancer”. Meanwhile, the down-regulated proteins were annotated to 27 major pathways, and the most enriched ones were “cell adhesion molecules”, “pathways in cancer” and “phagosome” (Figure 4A). The 19 highest ranked biological functions for the DEPs set are shown in Figure 4B. Among these pathways, “cell adhesion molecules” was enriched in ICOS ligand, poliovirus receptor, platelet endothelial cell adhesion molecule, cadherin-2, tumour necrosis factor receptor superfamily member 5, receptor-type tyrosine-protein phosphatase mu, cadherin-5, contactin-1, mucosal addressin cell adhesion molecule 1, CD276 antigen, vasorin, neuronal growth regulator 1, neogenin, golgi apparatus protein 1, junctional adhesion molecule C, cell adhesion molecule one and neurexin-3 (Supplementary Table S8).

### Eukaryoyic orthologous groups annotation, subcellular localisation and protein-protein interaction of differential expressed proteins

The potential functions of the identified DEPs were predicted using KOG, a database for the classification of protein orthologs (Supplementary Figure S3). The most representative KOG category was ‘cell processes and signalling’, which indicated that the DEPs were closely associated with signal transduction mechanisms, posttranslational modification, protein turnover and chaperones. Meanwhile, WoLF PSORT software was used to predict the subcellular localisation of the identified DEPs (Supplementary Figure S4). The most represented structures were located in extracellular, plasma membrane compartments, intracellular and mitochondria.

**FIGURE 5:**
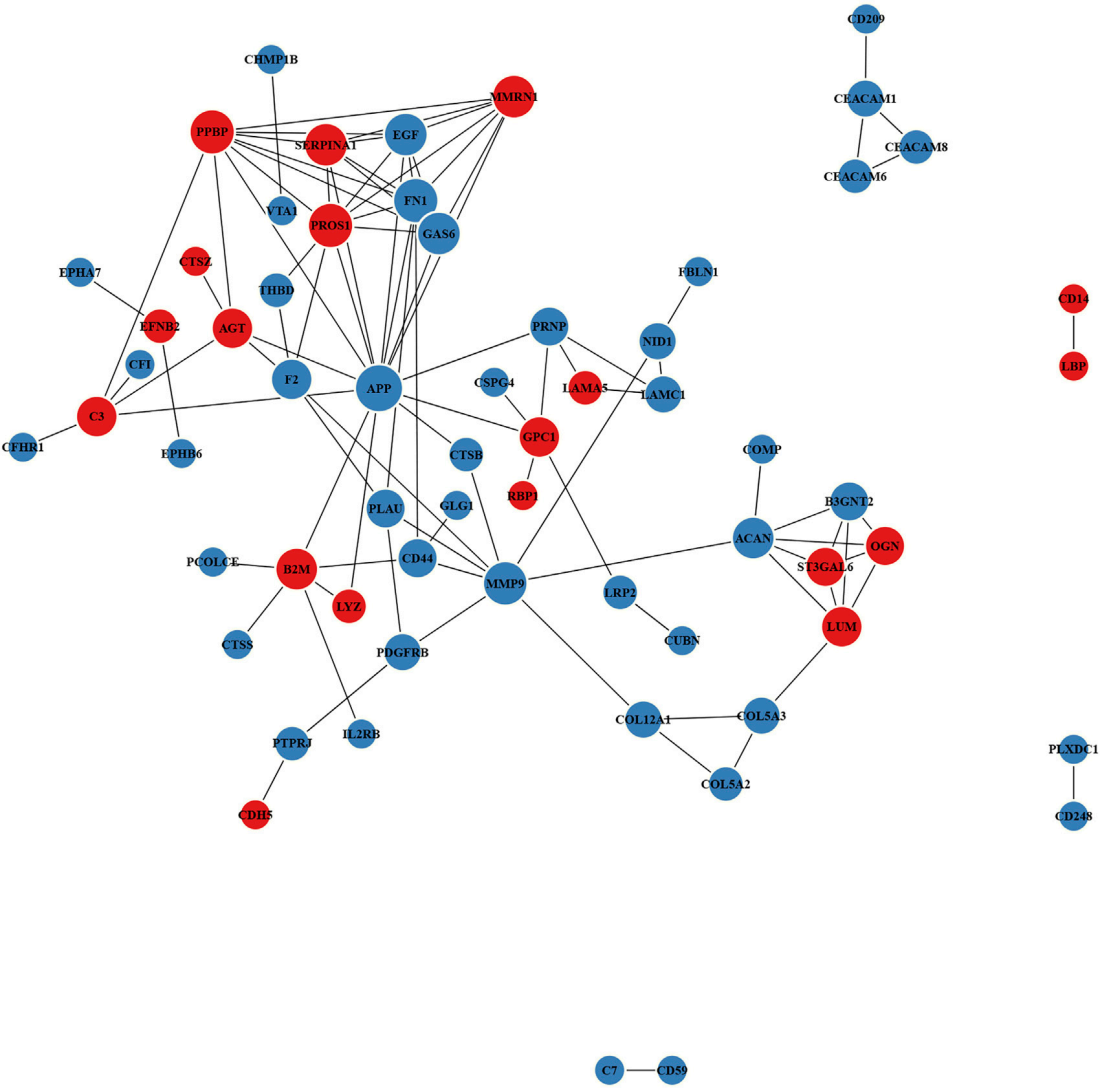
Protein–protein interaction (PPI) network of DEPs. Red and blue nodes separately represent up-regulated and down-regulated proteins. The size of the circles represents node degree.

Proteins often carry out a specific function after combining with a complex through PPI. For the top 100 confidence intervals, the STRING database was used for network interaction analysis of the DEPs to construct protein–protein relationships (Figure 5 and Supplementary Table S9). The hub eight central nodes were identified as vitamin K-dependent protein S (PROS), α-1-antitrypsin (A1AT), aggrecan core protein (PGCA), β-2-microglobulin (B2MG), AGT, matrix metalloproteinase-9 (MMP-9), growth arrest-specific protein 6 (GAS6) and urokinase-type plasminogen activator (PLAU). Among these central nodes, A1AT, B2MG, AGT and PROS belonged to the up-regulated DEPs, and the down-regulated DEPs included GAS6, MMP-9, PLAU and PGCA.

## Discussion

This study aimed to generate a high-quality resource of urinary proteomic datasets from patients with CSA-AKI and compare the alterations of urinary proteome profiles to reflect the renal status. The use of MS-based proteomic strategy provides a fresh perspective to understand the pathway mechanisms of CSA-AKI by determining the changes in the signal pathways of protein processing in the endoplasmic reticulum and cell adhesion molecules in patients with CSA-AKI. Gemini C18 column belongs to the new generation silica-based hybrid column, which introduces saturated hydrocarbons on the particle surface (Benhaim Grushka, 2008; Samuelsson et al., 2007). For the stationary phase of the Gemini C18 column, saturated carbons are inserted into the silica surface to form superhydrophobic systems, which can be used to characterise acidic and basic molecules in their neutral form due to the pH stability. Such a superhydrophobic surface can expand the pH range (pH 2–12) and maintain the superior column efficiency and mechanical strength of silica gel particles. In this case, the basic peptides hydrolysed by trypsin can be efficiently separated and enriched in the alkaline mobile phase (Supplementary Figure S5), providing abundant peptide sequencing information for DDA library construction and DIA quantitative proteomics. Additionally, Orbitrap Exploris HRMS has ultrahigh sensitivity and can accurately determine the composition of peptide ions. DIA quantitative proteomics has relatively high detection efficiency for low-abundance proteins and is suitable for exploring the relative expression changes of urinary proteins before and after AKI (Zi et al., 2014). Therefore, the use of enriched functional materials and Orbitrap HRMS can provide convenient conditions for the quantitative analysis of low levels of urinary proteins.

As a relatively common complication of cardiac surgery, AKI has an approximately 5%–42% incidence rate and has short-term and long-term survival implications for patients (Ostermann et al., 2021). Owing to the lack of effective AKI treatment methods, clinicians usually focus on prevention and risk factor management to reduce the incidence of AKI (Breilh et al., 2019; Goepfert et al., 2013; Khan et al., 2014). Thus, efforts have been exerted to identify biomarkers with high specificity and sensitivity relevant to CSA-AKI, including cardiac functional biomarkers, inflammation biomarkers and renal tubule-associated biomarkers (Wu et al., 2019). Global proteomic alterations in the sepsis-induced AKI model have also been investigated to reflect the systematic responses and identify a valuable resource for sepsis biomarker discovery (Lin et al., 2020). Patients with contrast-induced AKI also showed proteomic changes in urine, revealing the potential role of urinary proteomics in the assessment of early renal injury (Zhu et al., 2021). Therefore, the urinary proteome has become an important field for the discovery of non-invasive biomarkers and can also be used to distinguish subtle proteomic differences caused by specific diseases or therapeutic interventions.

KEGG analysis showed that “protein processing in endoplasmic reticulum” was the most enriched pathway for up-regulated DEPs, and “cell adhesion molecules” was the most enriched pathway for down-regulated DEPs. According to current evidence for ischaemia–reperfusion (IR) injury, endoplasmic reticulum (ER) stress has become an essential signal event of cell stress due to the accumulation of a large number of unfolded proteins in ER (Xu et al., 2016). These unfolded proteins might be eliminated *via* autophagy to alleviate the impact of protein misfolding in kidney diseases (Cybulsky, 2017). Therefore, ER stress may play a role in the pathogenesis of AKI, and early ER stress intervention and ER homeostasis restoration may help prevent or reduce the injury of renal cells (Tang et al., 2020). Cell adhesion molecules can mediate inflammatory processes through endothelial cells to amplify the immune response. The decrease in intercellular adhesion and the change of adhesion molecules may lead to renal function loss in AKI (Nürnberger et al., 2010). Furthermore, serum cell adhesion molecule levels, such as E-selectin levels, can be powerful predictors for early septic AKI (Su et al., 2016).

The PPI target networks of urinary DEPs were constructed using the STRING online database, and three hub DEPs with up-regulation and down-regulation were separately and comprehensively considered for AKI. Among the up-regulated DEPs, A1AT is a hepatic stress protein with protease inhibitor activity and can improve the binding efficiency with hemin to prevent the formation of hemin-induced reactive oxygen species in neutrophils (Zager et al., 2014). As a potential marker of neutrophil activation, serum A1AT can be used to predict AKI in patients with IR injury (Du et al., 2019). B2MG, a protein homologous to histocompatibility antigens, can be freely filtered into primary urine by the glomerulus. Owing to its increasing concentrations in the early stage of kidney failure, serum B2MG has already been proposed as a candidate biomarker to assess kidney function in AKI and CKD (Argyropoulos et al., 2017). Furthermore, the increased B2MG concentrations in urine have also been associated with hypoxia caused by cardiac surgery or kidney transplantation (Beitland et al., 2019). AGT is the parent polypeptide for the formation of angiotensin II, which can activate pro-inflammatory pathways in the renin–angiotensin–aldosterone system and may contribute to the progression of AKI (Ba et al., 2017). Clinical studies showed that urinary AGT can dynamically monitor the recovery of renal status after an AKI attack and may predict the progression of AKI-CKD and treatment response of patients with AKI (Chen C et al., 2016; Cui et al., 2018).

Among the down-regulated DEPs, GAS6 plays a role in leukocyte sequestration and migration, platelet aggregation and haematopoiesis, proliferation, apoptosis and phagocytosis as a member of the vitamin K-dependent protein family. Given that GAS6 is usually related to injury, inflammation and repair conditions, its contribution to AKI is closely associated with biological functions, including anti-apoptotic effect and survival-promoting capability (Xiao et al., 2021). GAS6 can exert protective roles by decreasing serum urea nitrogen, creatinine and renal apoptosis, reducing the sepsis-induced pathological damage and improving the survival rate of AKI mice (Chen L et al., 2016). MMP-9, a multi-domain zinc metalloproteinase released from inflammatory cells, can degrade the endothelial basement membrane and increase the permeability of capillaries (Bengatta et al., 2009). In addition to extracellular matrix remodelling, MMP-9 also regulates the activities of a variety of cytokines, receptors, chemokines, growth factors and cell adhesion molecules necessary for inflammation. The tissue inhibitor of matrix metalloproteinase-1 (TIMP-1) and MMP-9 can be used as potential diagnostic biomarkers for sepsis-associated AKI in clinical assessment (Bojic et al., 2015). As the soluble form of membrane-bound urokinase plasminogen activator receptor, PLAU is expressed in various cell types, such as neutrophils, monocytes, lymphocytes, endothelial cells and tumour cells (Hahm et al., 2017). Several clinical studies showed that PLAU has predictive and prognostic values in different kinds of AKI (Qin et al., 2021; Rasmussen et al., 2021). Thus, the present work can provide valuable knowledge on AKI-related urinary biomarkers for prediction and diagnosis of renal injury and signal pathways for potential therapeutic targets.

The major limitations of this study were the small sample size and fewer detection time points; hence, individual factors may affect the results of statistical analysis compared to the urinary metabolomics (Bai et al., 2022). A large number of patients in multicentre cohorts for rigorous algorithm analysis are needed in future studies of CSA-AKI urinary proteomics to minimise batch effects and confirm our results. Moreover, additional time points, such as constinuously recording kidney functional alteration before AKI, could facilitate the identification of differential substances and provide a new prospect for the discovery of potential biomarkers.

## Conclusion

The results indicated that renal signal pathways, including protein processing in ER and cell adhesion molecules, dramatically changed in patients with AKI. Such an MS-based proteomics strategy can help us obtain valuable resources to improve the understanding of the significant DEPs and pathway mechanisms of patients with CSA-AKI. This study will broaden our understanding of urinary proteome profiles reflecting the kidney status, promote the development of renal disease biomarkers and provide new insights into preventive treatments for CSA-AKI.

## Data Availability

The datasets presented in this study can be found in online repositories. The names of the repository/repositories and accession number(s) can be found in the article/Supplementary Material.
