# Supplementary material for: Urinary proteome analysis of acute kidney injury in post-cardiac surgery patients using enrichment materials with high-resolution mass spectrometry
Source: Front Bioeng Biotechnol. 2022 Sep 13;10:1002853. doi: 10.3389/fbioe.2022.1002853 (PMC9513377; doi:10.3389/fbioe.2022.1002853)
Supplement: Supplementary file 3 [file Table1.docx]

**Supplementary Materials**

**Table S1.** Clinical Characteristics of Six AKI Patients.

| Characteristics | Patients (n=6) |
| --- | --- |
| Age, y | 54.0±16.8 |
| BMI (kg/m^2^) | 20.6±4.7 |
| Smoke | 6 (100) |
| History |  |
| Hypertension | 1(16.7) |
| Diabetes | 0(0.0) |
| Hyperlipemia | 0(0.0) |
| coronary heart disease | 0(0.0) |
| Chronic kidney disease | 0(0.0) |
| RBC (10^9/L) | 5.0±0.5 |
| Hemoglobin (g/L) | 136.0±18.4 |
| Hematocrit level (%) | 41.5±3.8 |
| Albumin (g/L) | 37.8±4.1 |
| APACHE II score | 12.2±2.6 |
| Serum creatinine (μmol/L) | 99.9±22.1 |
| Glomerular filtration rate (ml/min) | 50.8±16.0 |
| Amount of blood loss (ml) | 291.7±20.4 |
| Duration of surgery (days) | 438.3±104.8 |
| Stay in hospital (days) | 29.2±11.2 |
| Length of ICU stay (days) | 2.2±0.8 |

**Table S2**. Construction of Peptide Library using DDA method.

**Table S3**. Summary of Peptide Identification from AKI patients’ samples.

**Table S4**. Summary of Protein Identification from AKI patients’ samples.

**Table S5**. Annotated Information of AKI Patients’ Urinary Proteome.

**Table S6**. Functional GO enrichment analysis of urinary DEPs.

**Table S7**. KEGG enrichment analysis of the identified urinary DEPs.

**Table S8**. Significant enrichment pathways of biological functions for the DEPs.

**Table S9**. PPI network analysis of DEPs.

**Figure S1.** Basic statistical charts for urinary protein identification. (A) unique peptide distribution; (B) protein mass distribution; (C) protein coverage distribution.

**Figure S2.** Functional GO classification of urinary proteins. (A) all the identified proteins; (B) DEPs.

**Figure S3.** KOG functional annotation of DEPs.

**Figure S4.** Subcellular localization of DEPs. The x-axis represents subcellular structure entries and y-axis represents the number of DEPs.

**Figure S5**. The interaction mechanisms with trypsin hydrolyzed peptides with Gemini C18 silica microspheres in the basic mobile phase.

**Data availability**

Mass spectrometry data have been deposited to iProX (ww.iprox.org) with ID IPX0004214000 and the ProteomeXchange Consortium ([http://proteomecentral.proteomexchange.org](http://proteomecentral.proteomexchange.org/)) *via* the iProX partner repository with the dataset identifier PXD032344.
