# Supplementary figures and images for: Urinary proteome analysis of acute kidney injury in post-cardiac surgery patients using enrichment materials with high-resolution mass spectrometry
Source: Front Bioeng Biotechnol. 2022 Sep 13;10:1002853. doi: 10.3389/fbioe.2022.1002853 (PMC9513377; doi:10.3389/fbioe.2022.1002853)

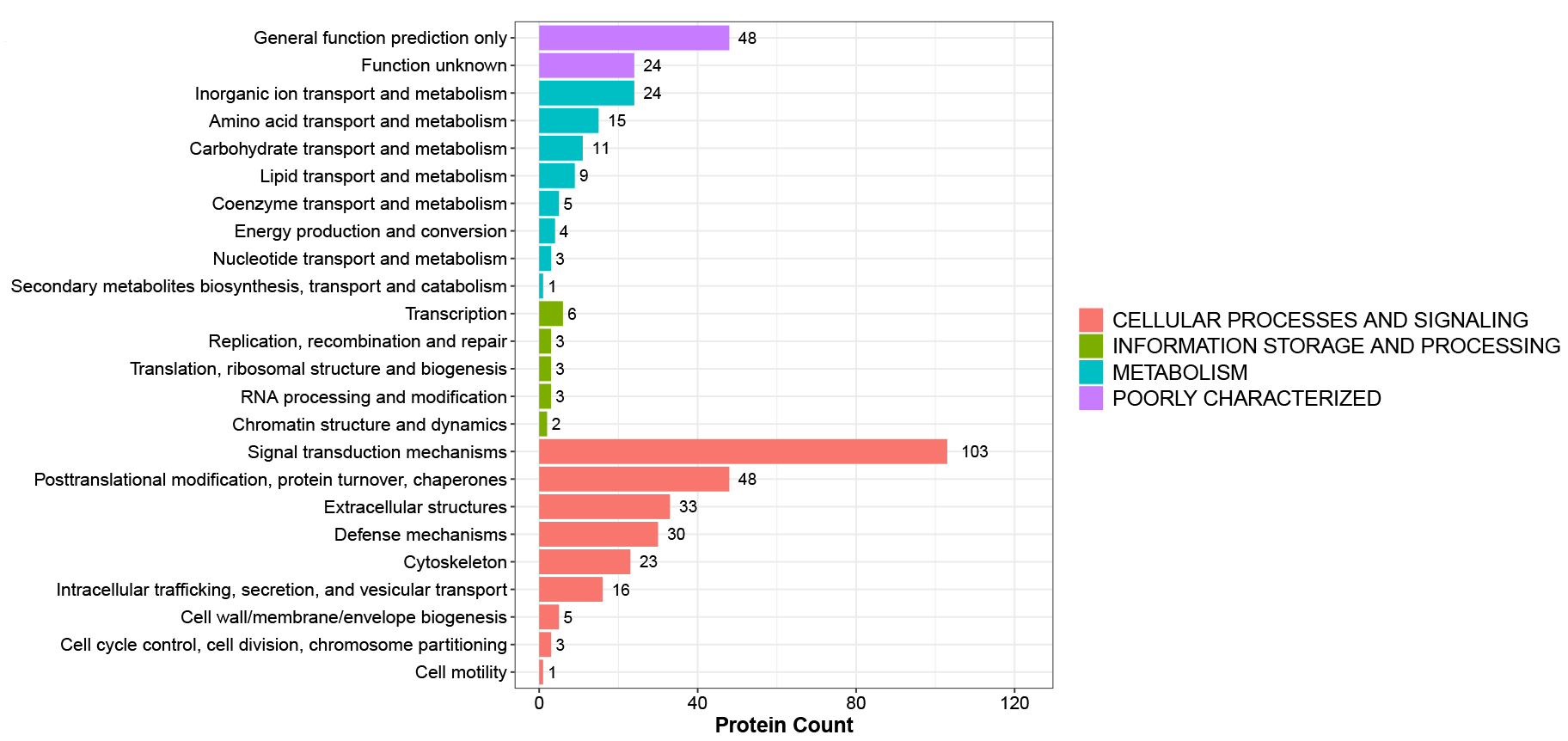

Supplement: Supplementary file 4 [file Image3.TIF]

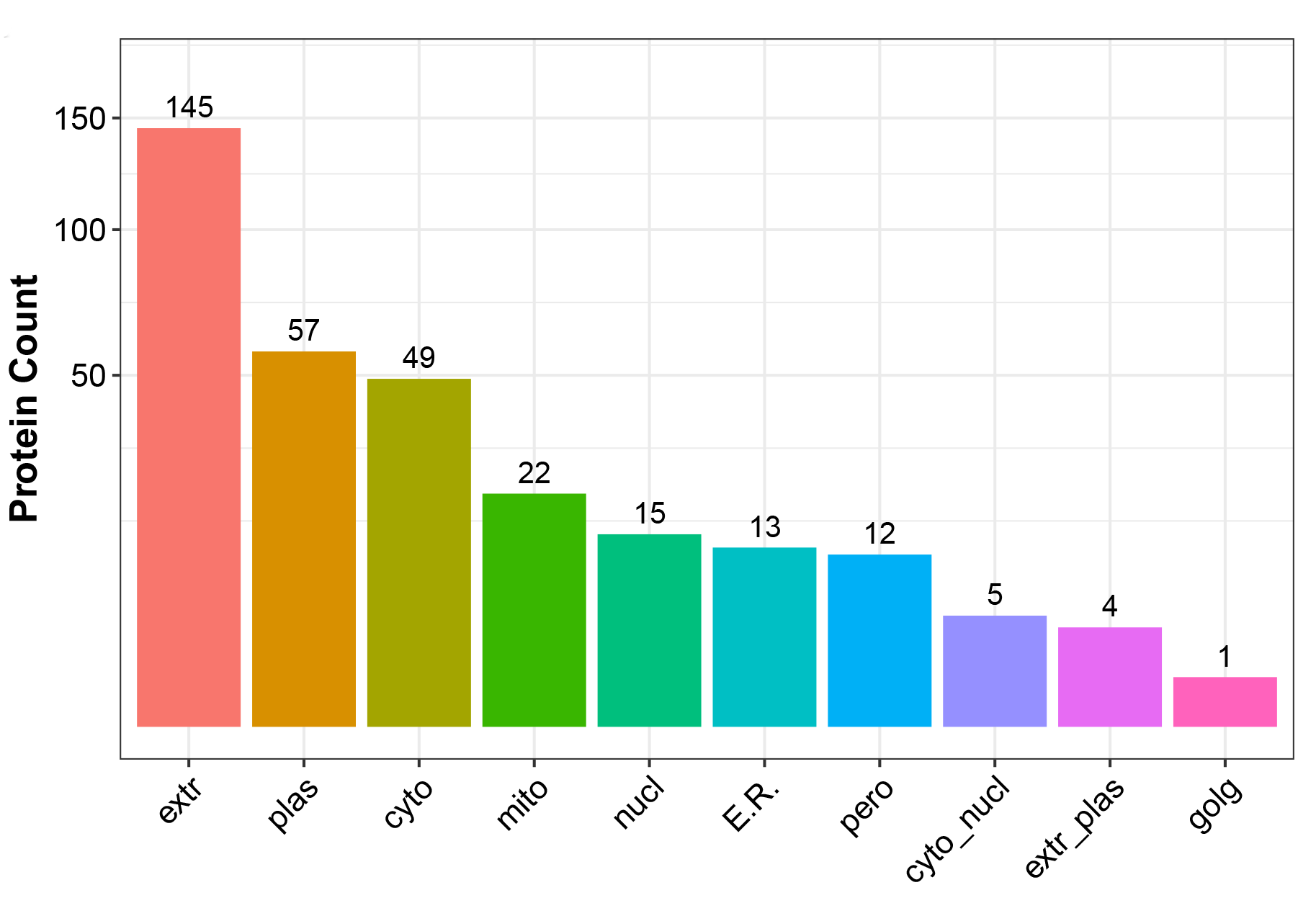

Supplement: Supplementary file 5 [file Image4.TIF]

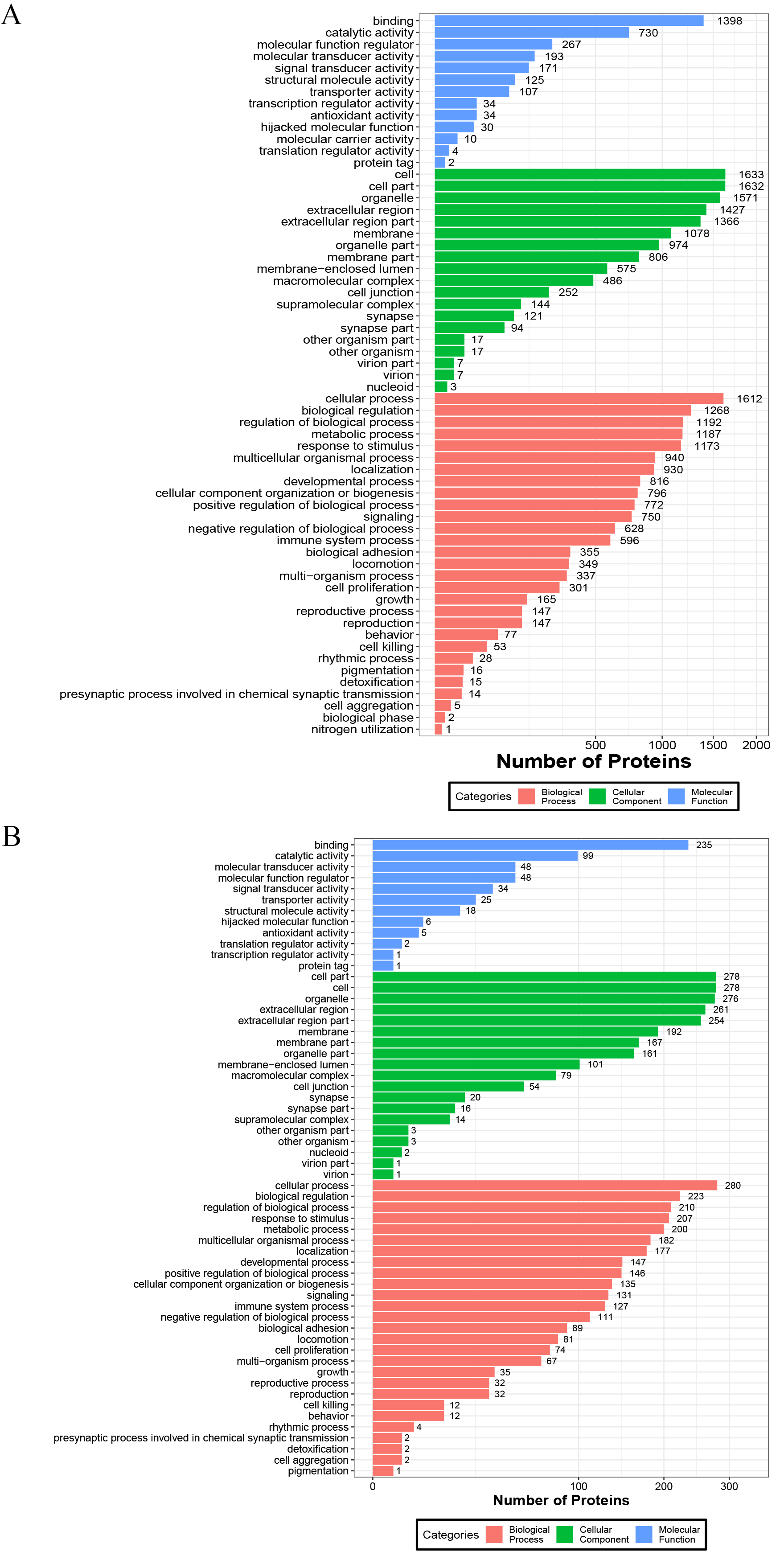

Supplement: Supplementary file 7 [file Image2.TIF]

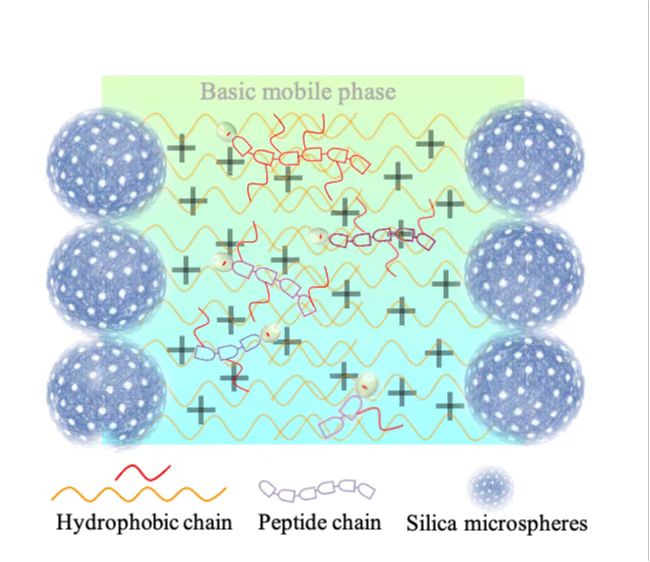

Supplement: Supplementary file 8 [file Image5.JPEG]

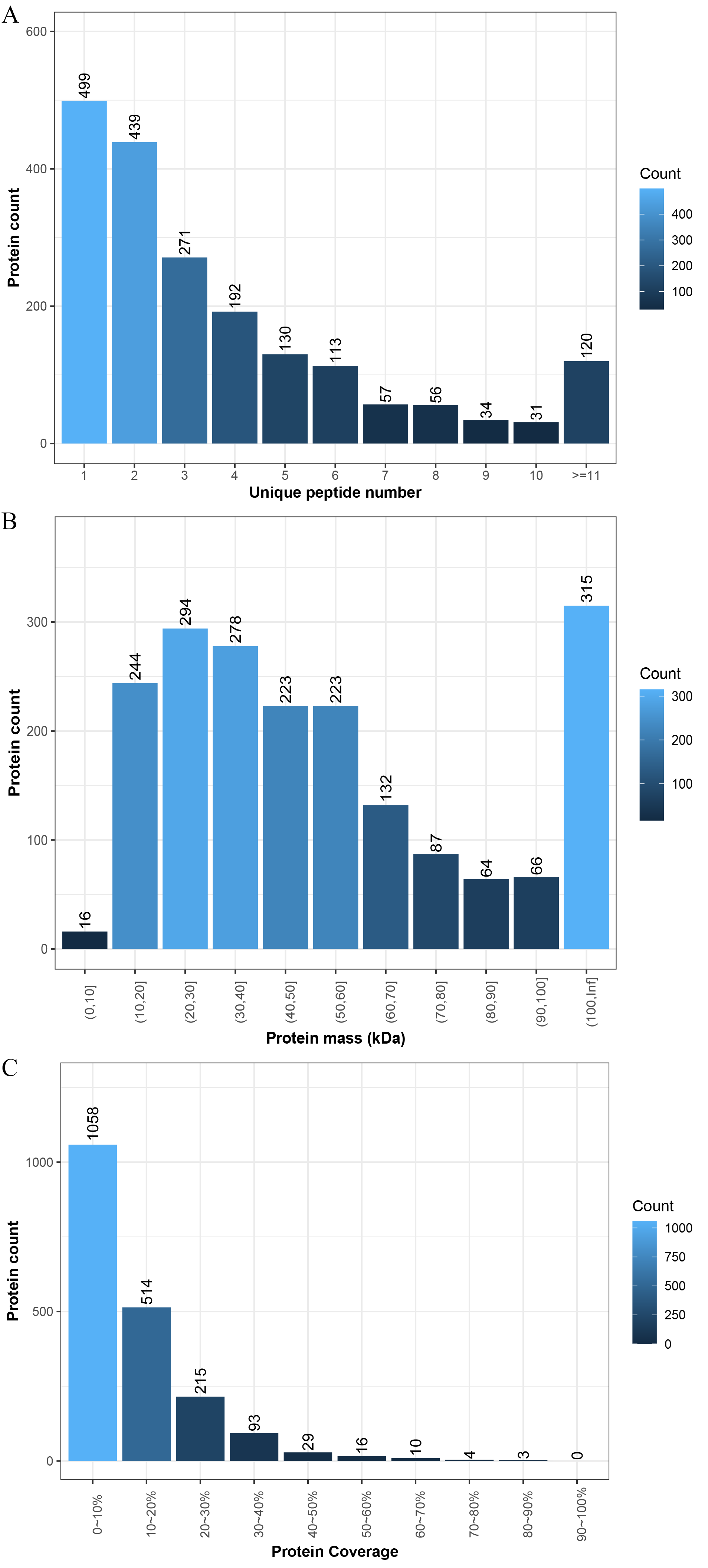

Supplement: Supplementary file 10 [file Image1.TIF]
